# Supplementary material for: A Biomimetic DNA‐Based Membrane Gate for Protein‐Controlled Transport of Cytotoxic Drugs
Source: Angew Chem Int Ed Engl. 2020 Nov 24;60(4):1903–8. doi: 10.1002/anie.202011583 (PMC7894144; doi:10.1002/anie.202011583)
Supplement: Supplementary file 1 — Supplementary [file ANIE-60-1903-s001.pdf]

Angewandte Chemie
Eine Zeitschrift der Gesellschaft Deutscher Chemiker

Supporting Information

A Biomimetic DNA-Based Membrane Gate for Protein-Controlled Transport of Cytotoxic Drugs

*Conor Lanphere[+], Patrick M. Arnott[+], Sioned Fôn Jones, Katarina Korlova, and Stefan Howorka**

anie_202011583_sm_miscellaneous_information.pdf

1. EXPERIMENTAL SECTION 2

1.1. Materials 2

1.2. DNA assembly 2

1.3. SDS PAGE 2

1.4. Thrombin-TBA electrophoretic mobility shift assay 2

1.5. Thrombin-pNP electrophoretic mobility shift assay 2

1.6. Lid-opening FRET assay 2

1.7. Preparation of fluorophore-filled LUVs and dye release assay 2

1.8. Cell culture 3

1.9. Cell-based assay 3

2. SUPPLEMENTARY TABLES 4

Table S1. Names, modifications and sequences of DNA oligonucleotides used for folding protein-gated nanopore (pNP) and variants. 4

Table S2. Names and strand compositions of DNA nanopores 5

3. SUPPLEMENTARY FIGURES 5

Figure S1. 2D DNA maps of protein-gated nanopore pNP, and variants pNP2 and pNP^{TAMRA}. 5

Figure S2. Top-down and side view of closed pNP. 6

Figure S3. Gel electrophoretic shift assay to quantify the binding of thrombin to the TBA aptamer carrying a 26-nucleotide tail. 6

Figure S4. Formation of the protein-gated nanopore pNP and binding to membrane vesicles. 7

Figure S5. Gel electrophoretic shift assay to quantify the binding of thrombin to pNP2. 7

Figure S6. Fluorescence analysis of the thrombin-triggered opening of pNP. 8

Figure S7. Kinetic fluorescence analysis on the thrombin-triggered opening of pore variant pNP2. 8

Figure S8. Kinetic fluorescence analysis on the requirement of a matching aptamer sequence for protein-triggered channel opening. 9

Figure S9. Dynamic light scattering analysis LUVs with encapsulated sulforhodamine B. 9

Figure S10. Thrombin-mediated opening of pNP2 controls transport of molecular cargo across lipid bilayers 10

Figure S11. Comparison of the thrombin-mediated SRB transport properties of pNP and pNP2 from vesicles. 10

Figure S12. Dye transport assay investigating the thrombin-triggered fluorophore release as a function of pNP or pNP2 concentration. 11

Figure S13. Comparison of SRB dye release from vesicles carrying pNP or pNP3. 12

Figure S14. Brightfield images of HeLa cells, treated with different components of the nanodevice. . 13

1. Experimental Section

1.1. Materials

Unmodified, fluorophore-labelled and cholesterol-modified DNA oligonucleotides were purchased from Integrated DNA Technologies on a 100 nmol scale with HPLC purification. 1,2-dioleoyl-*sn*-glycero-3-phosphocholine (DOPC), 1,2-dioleoyl-*sn*-glycero-3-phosphoethanolamine (DOPE) and 1-palmitoyl-2-oleoyl-*sn*-glycero-3-phosphocholine (POPC) were procured from Avanti Polar Lipids (US). Human alpha-thrombin was purchased from Haematologic Technologies on a 100 µg scale in 50% glycerol/water (v/v). All other reagents and solvents were purchased from Merck (UK) unless specified.

1.2. DNA assembly

Equimolar mixtures of DNA oligonucleotides (1 µL each, stock concentration of 100 µM)(Table S2 for composition of DNA pores) were dissolved at 1 µM in a buffer solution of 12 mM $MgCl_2$ in 0.6x TAE (40 mM Tris, 20 mM acetic acid), pH 7.4 to a final volume of 100 µL. Folding was achieved on a BioRad PCR thermocycler (UK) using a programme including heating to 85°C for 10 min, cooling to 65°C within 5 min, cooling to 25 °C at a rate of 0.1 °C per 2 min, and cooling to 10 °C at a rate of 0.2 °C per min. Samples were stored at 4 °C.

1.3. SDS PAGE

The assembled DNA nanostructure and component DNA oligonucleotides were analysed with commercial 10% polyacrylamide gels (BioRad, UK) in 1x TGS (25 mM Tris, 192 mM glycine and 0.1% SDS, pH 8.6). For gel loading, a solution of the DNA nanopores (2 µL, 1 µM) was mixed with folding buffer (13 µL, 2 mM $MgCl_2$ in 0.6x TAE, pH 7.4) and 6x gel loading dye (5 µL, New England Biolabs, UK). The gel was run at 60 V for 60 min at 4°C. The gel bands were visualised by staining with ethidium bromide and UV illumination. A 100 bp marker (New England Biolabs, UK) was used as a reference standard.

1.4. Thrombin-TBA electrophoretic mobility shift assay

TBA (10 µL, 4 µM) were mixed with thrombin (10 µM stock) in buffer A (0.3 M KCl, 15 mM Tris pH 7.4) yielding concentrations of 0 to 2.67 µM in a final volume of 50 µL. After incubation for 30 min at 30°C, and 6x gel loading dye (10 µL, New England Biolabs) was added, and the samples were loaded onto a thermally equilibrated 2% agarose gel. The gel was run in 1x TAE buffer, pH 8.3 at 60 V for 60 min at 4°C. Staining and molecular markers were as described above.

1.5. Thrombin-pNP electrophoretic mobility shift assay

pNP (2 µL, 1 µM) was mixed with thrombin (10 µM) in buffer A yielding concentrations of 0 to 3.2 µM in a final volume of 20 µL. After incubation for 30 min at 30°C, 6x gel loading dye (5 µL) was added, and the samples were loaded onto thermally equilibrated 10% PAGE. The gel was run in 1x TBE buffer (89 mM Tris-borate and 2 mM EDTA, pH 8.3) at 60 V for 90 min at 4°C. Staining and molecular markers were as described above.

1.6. Lid-opening FRET assay

The extent of the protein-gated lid opening of pNP was determined by monitoring Cy3 emission (ex_{540nm}, em_{570nm}) using a fluorescence spectrophotometer (Cary Eclipse, Agilent, UK). To a quartz cuvette (Hellma Analytics), pNP (11 µL, 1 µM) were added to buffer A (108.5 µL or 93.5 µL) and, after 5 min incubation, thrombin in buffer A (40 µM) was added (1.5 µL or 7.5 µL) yielding the final concentrations of 0.2 or 2 µM equivalent to pNP:thrombin ratios of 1:2 or 1:20, respectively. Lid opening was monitored for 40 min.

1.7. Preparation of fluorophore-filled LUVs and dye release assay

Solutions of the lipids DOPC (70 µL, 10 mM) and DOPE (30 µL, 10 mM) in chloroform were added to a 5 mL round bottom flask. The solvent was removed using a rotary evaporator (Buchi) to yield a thin film,

which was further dried under high vacuum (Buchi) for 1 h. The lipid was re-suspended in PBS (137 mM NaCl, 2.7 mM KCl, 10 mM Na_2HPO_4, 1.8 mM KH_2PO_4, pH 7.4) containing SRB (50 mM). The solution was sonicated for 20 min at 30°C and then equilibrated for 3 h. LUVs were extruded 25 times through a 200 nm polycarbonate membrane (Avanti Polar Lipids, US) using the extruder kit (Avanti Polar Lipids, US). The non-encapsulated dye was removed using a NAP-25 column (GE Healthcare, UK), and LUVs were exchanged into buffer B (0.2 M KCl, 10 mM Tris pH 7.4). LUVs were then subjected to dynamic light scattering with a Malvern Zetasizer Nano S (UK) to confirm the vesicles' diameter. Purified LUVs were used within 48 h and gently resuspended immediately prior to use. For release assays, the LUV suspension with encapsulated SRB (10 µL), pNP (30 µL, 1 µM) and buffer B (95 µL, 102.5 µL, 108.5 µL) were added to a 10 mm quartz cuvette (Hellma Analytics). Fluorescence was monitored at 586 nm and excited at 565 nm. After 5 min, thrombin (15 µL, 7.5 µL, 1.5 µL; 20 µM in buffer A) was added to give a ratio of 1:1, 1:5 or 1:10 (pNP:thrombin) in final volume of 150 µL. After 60 min of monitoring fluorescence, samples were mixed with a 1% solution of Triton X-100 (10 µL) to lyse all vesicles to identify maximum SRB release. Maximum fluorescence emission and the fluorescence prior to addition of thrombin were used to calculate the kinetics of release as %. For the kinetic analysis of efflux, the first 5 min of three 2 µM thrombin-mediated release traces were fitted with a linear line-of-best-fit. For the 0 µM thrombin-mediated release trace (baseline), the first 10 min of seven traces obtained from different batches of fluorophore-filled LUVs were averaged to one trace which was fitted to the line-of-best-fit. From these lines-of-best-fits, the initial rate at 1 min, 1.5 min, and 2 min were calculated and averaged to minimize fluctuations in the data.

1.8. Cell culture

HeLa cells were a gift from Dr Jeremy Carlton, King's college London. Cells were cultured at 37 °C and 5% CO_2 in Dulbecco's Modified Eagle Medium (DMEM) GlutaMAX™ (Gibco; Life Technologies) supplemented with 10% heat-inactivated Fetal Bovine Serum (FBS) (Gibco; Life Technologies) and 1% Penicillin – Streptomycin solution (Gibco; Life Technologies).

1.9. Cell-based assay

On day 0, HeLa cells were plated at a density of 10,000 cells per well in a 96-well plate and left to grow overnight. The next day (day 1), cells were supplanted with fresh culture medium and treated with either: thrombin (10 µL, 165 µM) in buffer A, pNP2 (10 µL, 1 µM); topotecan (10 µL, 3 µM), LUVs filled with 3 µM topotecan (10 µL, 0.1 mM, PC:PE 7:3 lipid ratio), pNP2 functionalized topotecan-filled LUVs (20 µL), and the latter in combination with thrombin (10 µL, 165 µM in buffer A). All wells were made up to final volume of 100 µL. The treated cells were maintained in a humidified atmosphere, containing 5% CO_2 at 37°C, for 3 d. Brightfield images were captured at day 1, 2 and 3 using an inverted Nikon Eclipse microscope, 20x air objective. Images were processed using ImageJ. On day 3, a WST-1 colorimetric assay was used to quantify cell viability. WST-1 (10 µL) was added to the culture medium in each well (100 µL)[55] and incubated for 3 h. The absorbance was determined at 450 nm using a microplate reader (VICTOR multilabel plate reader, PerkinElmer), and the absorbance reading at 620 nm was used as a reference. The experiment was performed in triplicate.

2. Supplementary Tables

Table S1. Names, modifications and sequences of DNA oligonucleotides used for folding protein-gated nanopore (pNP) and variants.

ID	Sequence 5′ → 3′
TBA	GGTTGGTGTGGTTGGTTTAAGTAGTTCAAGACCCCGTGACT
1	AGCGAACGTGGATTTTGTCCGACATCGGCAAGCTCCCTTTTTCGACTATT
2	CCGATGTCGGACATTCGCTGCGCGGTTTTTTTAAGTAATCACGTTCACGATC<br>TTCGCCTGCTGGGTTTTGGGAGCTTG
3	CGAAGATCGTGTTTTTCCACAGTTGATTGCCCTTCACTTTTCCCAGCAGG
4	AATCAACTGTGGTTTTTCTCACTGGTGATTAGAATGCTTTTGTGAAGGGC
5	TCACCAGTGAGATAGTCACGATATTTTGCACGTCATATTATGTCGTACCAGG<br>TGCATGGATTTTTGCATTCTAA
6	CCTGGTACGACATTTTTCCACGTTCGCTAATAGTCGATTTTATCCATGCA
1(chol)	Sequence of 1 carrying a cholesterol *via* tri(ethylene glycol) TEG linker at the 3′<br>terminus
3(chol)	Sequence of 3 carrying a cholesterol *via* a TEG linker at the 3′ terminus
4(chol)	Sequence of 5 carrying a cholesterol *via* a TEG linker at the 3′ terminus
6(chol)	Sequence of 5 carrying a cholesterol *via* a TEG linker at the 3′ terminus
lid	AACCGCGCAGCGTTTTTTTTTTATGACGTGCTTTTTTTTTTATCGTGACGGTTG<br>GTGTGGTTGGCGTGATTACTTA
lid(Cy3)	AACCGCGCAGCGTTTTTTTTTTATGACGTGCTTTTTTTTTTATCGTGACGGTTG<br>GTGTGGTTGGCGTGATTACTTA^{Cy3}
3(Cy5)	CGAAGATCGTGTT^{Cy5}TTTCCACAGTTGATTGCCCTTCACTTTTCCCAGCAGG
lid-II	AACCGCGCAGCGGGTTGGTGTGGTTGGTATGACGTGCTTTTTTTTTTATCGTG<br>ACTTTTTTTTTTCGTGATTACTTA
lid-II(Cy3)	ACCGCGCAGCGGGTTGGTGTGGTTGGT^{Cy3}ATGACGTGCTTTTTTTTTTATCGT<br>GACTTTTTTTTTTCGTGATTACTTA
lid-control	AACCGCGCAGCGTTTTTTTTTTATGACGTGCTTTTTTTTTTATCGTGACTTTTTT<br>TTTCGTGATTACTTA
lid-control(Cy3)	AACCGCGCAGCGTTTTTTTTTTATGACGTGCTTTTTTTTTTATCGTGACTTTTTT<br>TTTCGTGATTACTTA^{Cy3}
lid-II-ext	AACCGCGCAGCGGGTTGGTGTGGTTGGTATGACGTGCTTTTTTTTTTATCGTG<br>ACTTTTTTTTTTCGTGATTACTTACACCGATCAAGAAG
dye-ext	CTTCT^{T}TTGATCGGTG

X^{Cy3} = Cy3 fluorophore; X^{Cy5} = Cy5 fluorophore; X^{T} = TAMRA fluorophore.

Table S2. Names and strand compositions of DNA nanopores

Nanopore	Oligonucleotides used
pNP$^{\Delta C}$	1, 2, 3, 4, 5, 6, lid
pNP2$^{\Delta C}$	1, 2, 3, 4, 5, 6, lid-II
pNP3$^{\Delta C}$	1, 2, 3, 4, 5, 6, lid-control
pNP	1(chol), 2, 3(chol), 4(chol), 5, 6(chol), lid
pNP2	1(chol), 2, 3(chol), 4(chol), 5, 6(chol), lid-II
pNP3	1(chol), 2, 3(chol), 4(chol), 5, 6(chol), lid-control
pNP-L$^{\Delta C}$	1, 2, 3, 4, 5, 6
pNP^{TAMRA}	1(chol), 2, 3(chol), 4(chol), 5, 6(chol), lid-II-ext, dye-ext
pNP$^{\Delta C,Cy3,Cy5}$	1, 2, 3(Cy5), 4, 5, 6, lid(Cy3)
pNP2$^{\Delta C,Cy3,Cy5}$	1, 2, 3(Cy5), 4, 5, 6, lid-II(Cy3)
pNP3$^{\Delta C,Cy3,Cy5}$	1, 2, 3(Cy5), 4, 5, 6, lid-control(Cy3)

3. Supplementary Figures

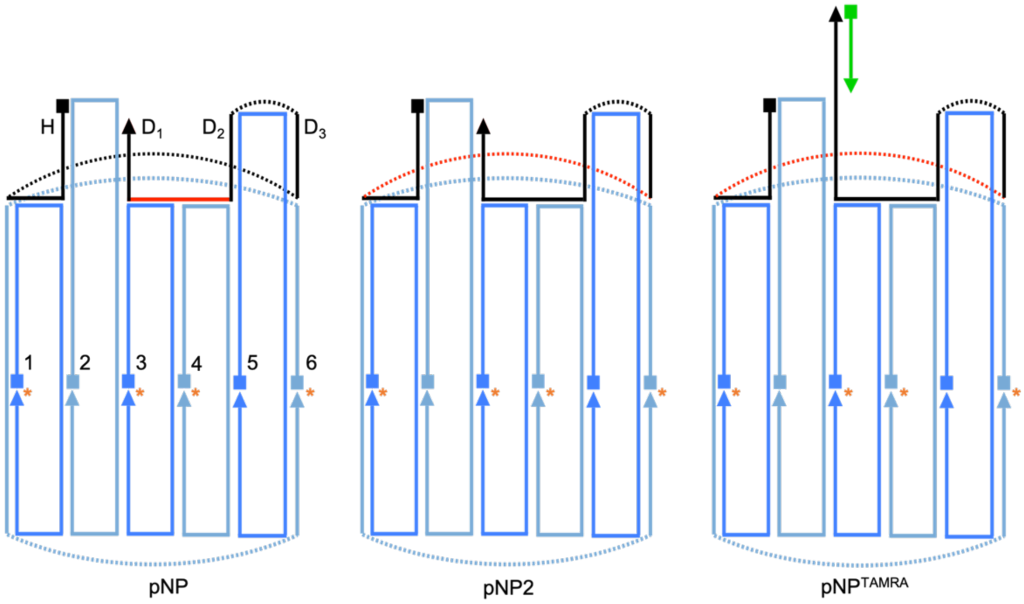

Figure S1. 2D DNA maps of protein-gated nanopore pNP, and variants pNP2 and pNP^{TAMRA}. Strands are labelled 1-6. Squares denote the 5' end of DNA and triangles the 3' end. Orange asterisks indicate the position of cholesterol anchors, the red coloured section represents the TBA sequence in the lid, Green indicates oligonucleotide dye-ext. carrying the TAMRA-dye, and H and D1-D3. indicate the hinge region of the lid that remains bound to the pore, and the docking regions, respectively.

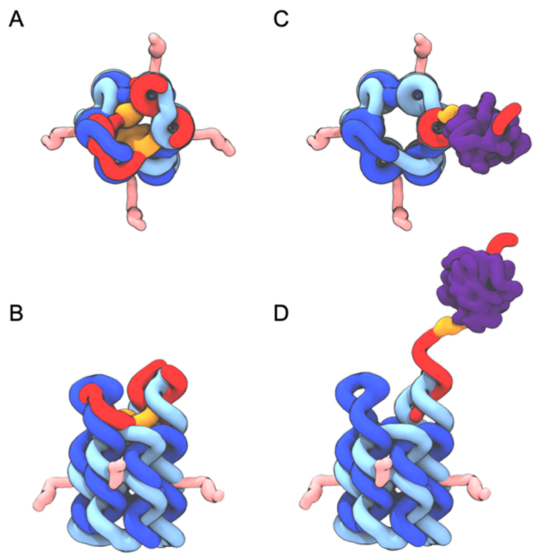

Figure S2. Top-down and side view of closed pNP.
The pore's barrel is composed of six component DNA strands (light and dark blue) that form six interconnected DNA duplexes arranged in hexagonal fashion. The protein-sensitive lid (red, orange) features the thrombin-binding aptamer (orange) and blocks the channel of pNP – see top-down (A) and side-view (B) of the closed pNP. Top-down (C) and side view (D) of pNP following binding to thrombin (dark purple), which leads to the partial dissociation of the lid and the opening of pNP's channel. Four cholesterol anchors (pink) insert pNP into the hydrophobic lipid bilayer membrane (not shown).

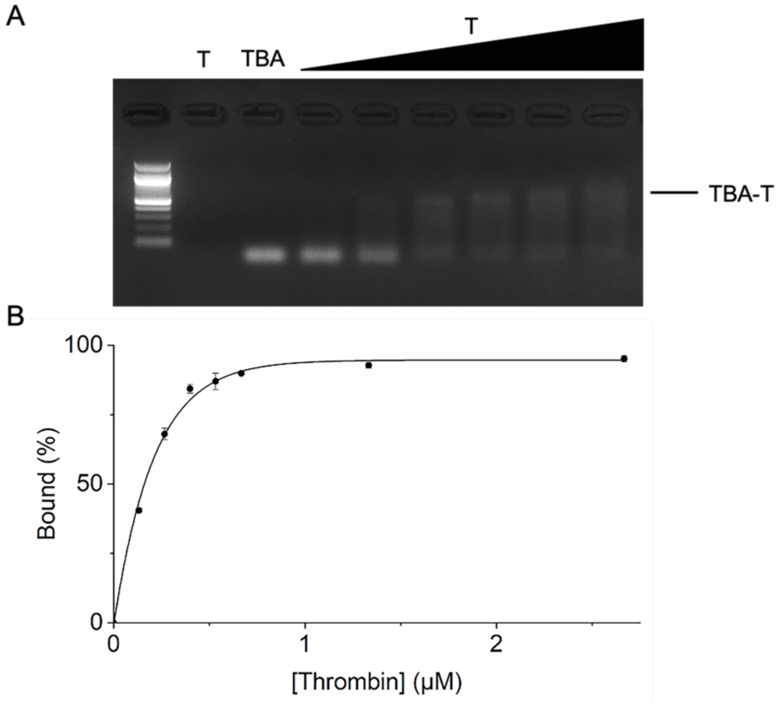

Figure S3. Gel electrophoretic shift assay to quantify the binding of thrombin to the TBA aptamer carrying a 26-nucleotide tail.
(A) Within the gel shift assay, TBA (0.67 μM) was mixed with increasing concentrations of thrombin (from 0 to 2.67 μM). The mixtures were analysed via 2% agarose gel electrophoresis. The weakening of the TBA band and the occurrence of an upshifted band indicates the formation of a TBA-T complex. (B) Plot of the relative amount of TBA bound to thrombin as a function of thrombin concentration. The data represent averages and standard deviations from 3 independent experiments.

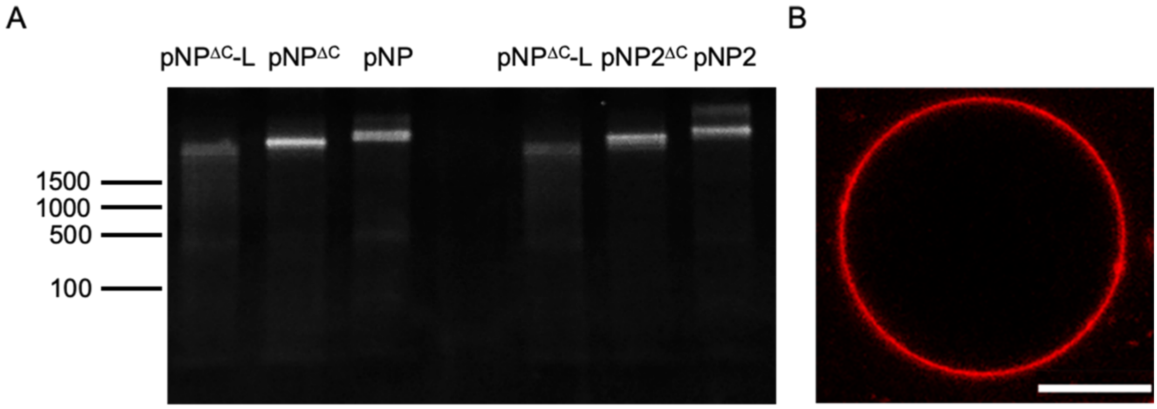

Figure S4. Formation of the protein-gated nanopore pNP and binding to membrane vesicles.
(A) Assembly of pNP and pNP2 was confirmed by 10% SDS PAGE. Lanes from left to right: pNP-L, pNPΔC, pNP, pNP-L, pNP2ΔC, pNP2, the annotation on the left represents specified 100 bp DNA ladder bands. (B) CLSM shows binding of fluorophore-labelled pNPTAMRA to POPC giant unilamellar vesicles, scale bar 10 μm.

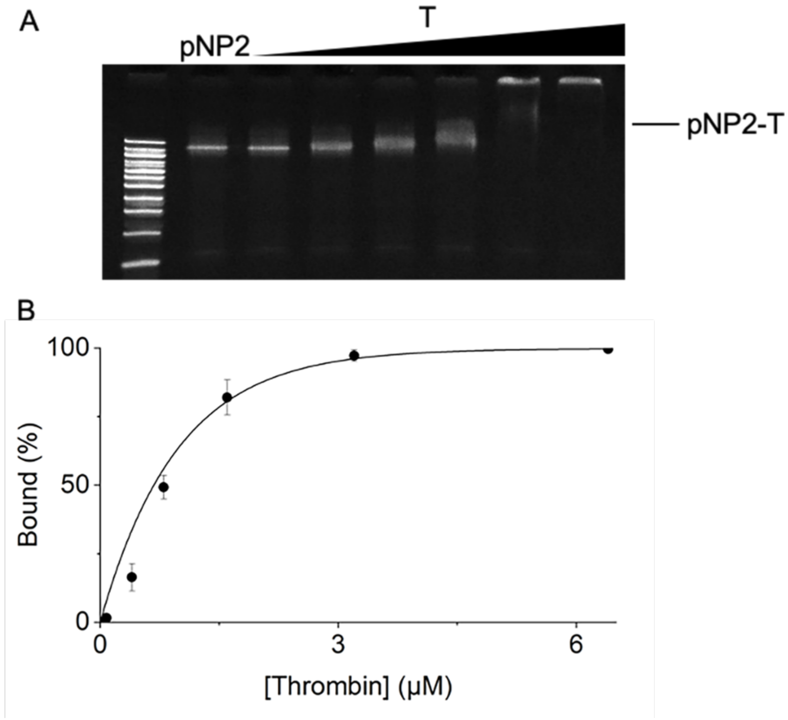

Figure S5. Gel electrophoretic shift assay to quantify the binding of thrombin to pNP2.
(A) Within the gel shift assay, pNP2 (0.08 μM) was mixed with increasing concentrations of thrombin (T, from 0 to 6.4 μM). Electrophoretic analysis showing that increasing the concentration of thrombin leads to the disappearance of the pNP2 band and the appearance of an upshifted band representing the pNP2-thrombin complex (pNP2-T). (B) Plot of the relative amount of pNP2 bound to thrombin as a function of thrombin concentration. The data represent averages and standard deviations from 3 independent experiments.

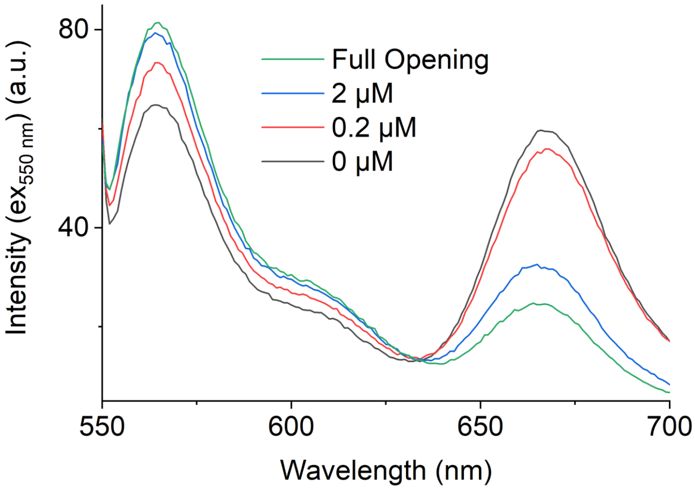

Figure S6. Fluorescence analysis of the thrombin-triggered opening of pNP.
The pore carries a Cy3 tag at the 3' end of the lid and a Cy5 tag at the pore opening. The close proximity of the two dyes leads to reduced Cy3 emission in the pore's closed state. Mixing pNP (0.1 µM) with thrombin to a final concentration of 0.2 µM (red) and 2 µM (blue) causes the protein-triggered opening of the lid of the pNP and the separation of the dyes and increased Cy3 emission. No increase in emission was observed in the absence of thrombin (black) while full opening (green) was observed after heating the pore to 55˚C to thermally open the lid. The increase in Cy3 emission indicates that thrombin binding leads to the lid's partial unzipping from the pore thereby separating the Cy3-lid from the Cy5 at the gate's barrel.

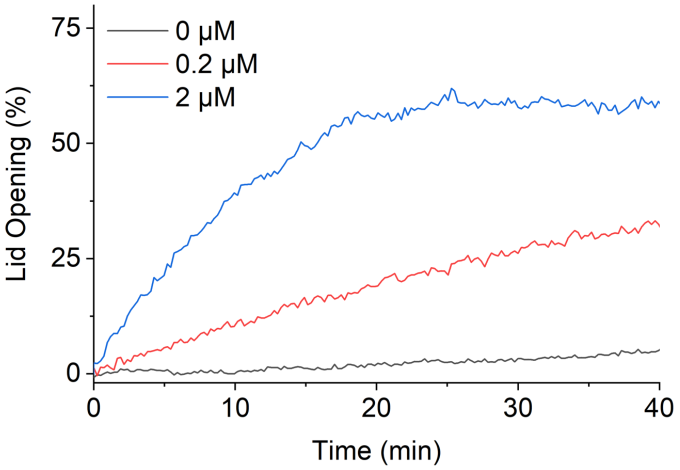

Figure S7. Kinetic fluorescence analysis on the thrombin-triggered opening of pore variant pNP2.
The pore carries a Cy3 tag at the 3' end of the lid and a Cy5 tag at the pore opening. The close proximity of the two dyes leads to the quenching of Cy3 emission in the pore's closed state. Mixing pNP2 (0.1 µM) at time-point 0 min with thrombin to final concentrations of 0.2 µM (red) and 2 µM (blue) leads to a gradual increase of Cy3-emission indicating the protein-triggered lid opening. The data are normalized to complete opening obtained by the heating of pore to 55˚C to thermally open the lid. No increase in emission was observed in the absence of thrombin (black).

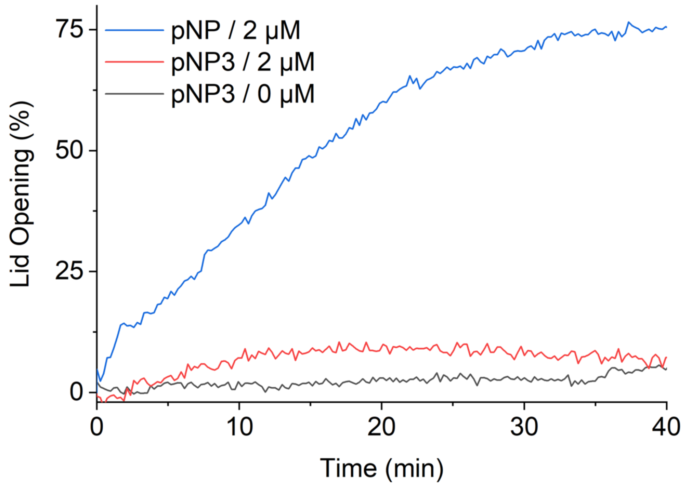

Figure S8. Kinetic fluorescence analysis on the requirement of a matching aptamer sequence for protein-triggered channel opening.
The variant pNP3 lacks the TBA sequence in the lid. Cy3 emission from the lid is quenched by the Cy5 attached to the top of the pore in its closed state. Addition of thrombin at 2 μM (red) to 0.1 μM pNP3 does not lead to significant lid opening, as indicated by the unchanged Cy3 emission similar to protein-free buffer (0 μM, black). As reference, the plot also includes the trace (blue) from the paper's Figure 2 for lid opening following addition of 2 μM thrombin to 0.1 μM pNP.

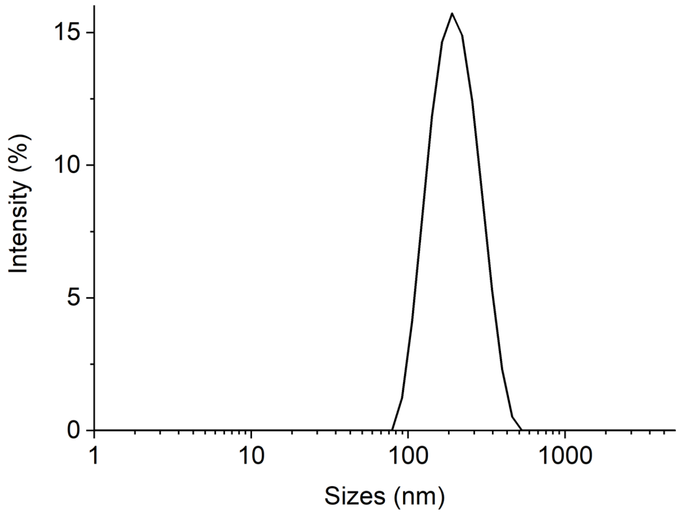

Figure S9. Dynamic light scattering analysis LUVs with encapsulated sulforhodamine B.
The analysis of three repeats revealed an average vesicle diameter of 216 ± 8 nm consistent with extrusion through a 200 nm polycarbonate membrane. Vesicles were made with a 7:3 mole ratio of DOPC:DOPE.

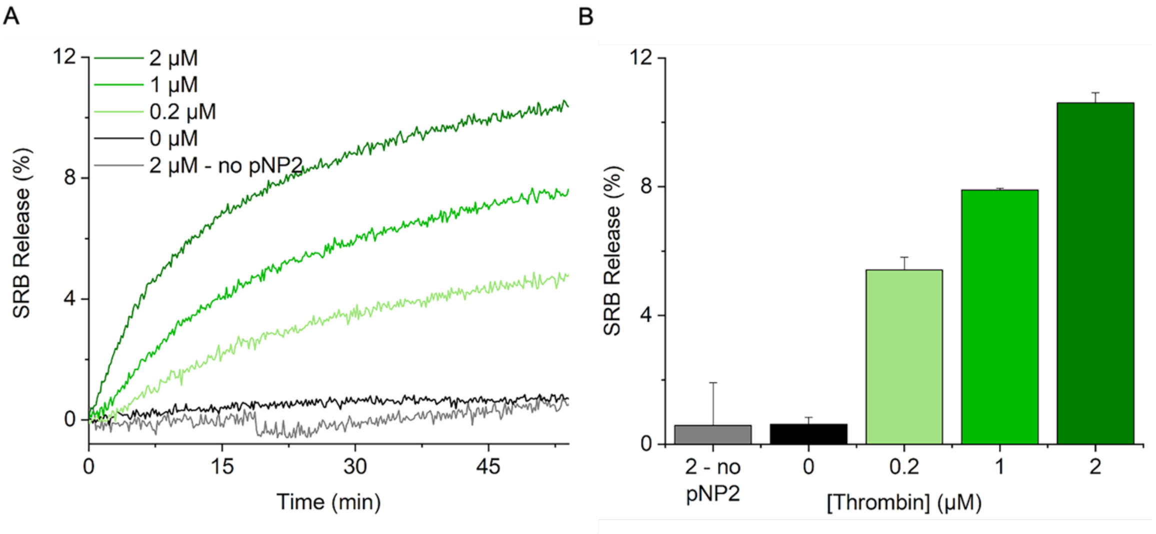

Figure S10. Thrombin-mediated opening of pNP2 controls transport of molecular cargo across lipid bilayers.

The experimental set-up is the same as in Figure 3 of the paper for pNP. In the assay, fluorophore sulforhodamine B (SRB) is self-quenched inside lipid vesicles. pNP2 is inserted within the vesicle membrane and can be opened upon addition of thrombin to release SRB from the vesicles leading to increased fluorescence. (A) Kinetic traces of SRB fluorescence as a function of increasing thrombin concentration. A maximum of 100% release is the total amount of fluorescence obtained upon rupturing vesicles with detergent Triton-X-100. (B) Bar chart showing the percentage increase in SRB emission from (A). The data represent averages and standard deviations from at least 3 independent experiments.

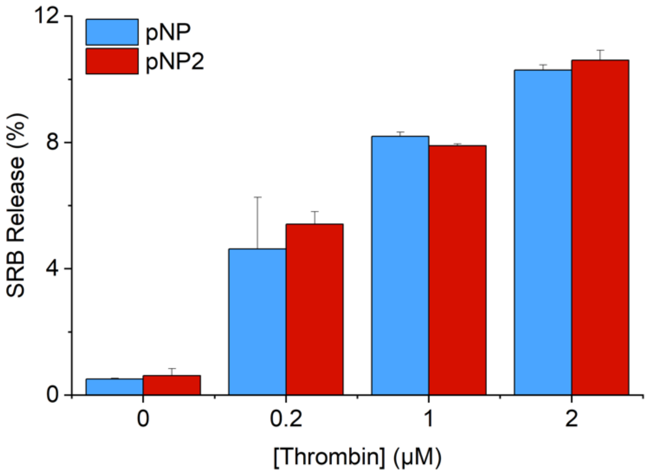

Figure S11. Comparison of the thrombin-mediated SRB transport properties of pNP and pNP2 from vesicles.

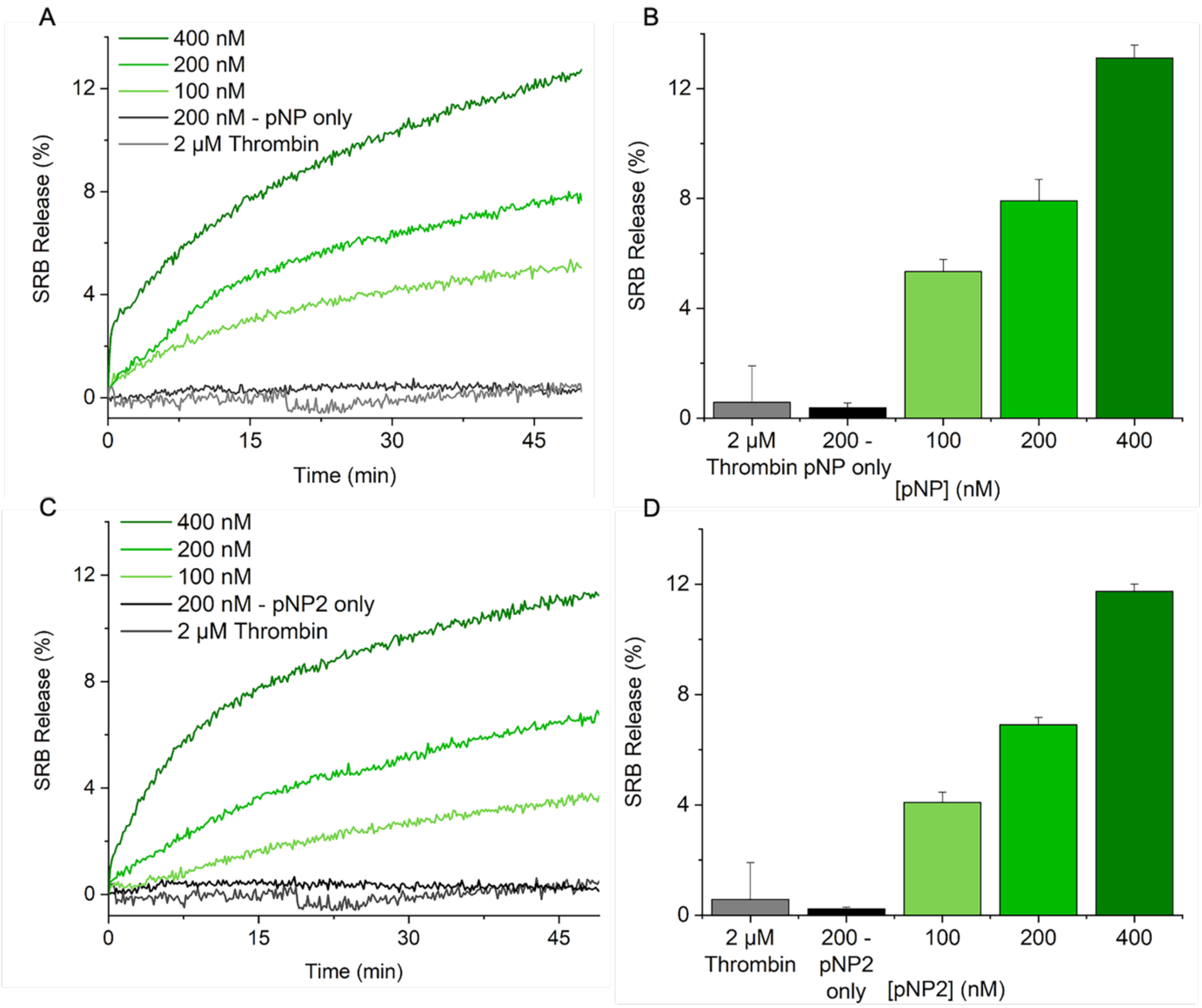

Figure S12. Dye transport assay investigating the thrombin-triggered fluorophore release as a function of pNP or pNP2 concentration.
The experimental set-up is the same as in Figure 3 of the paper. The ratio of thrombin:pore was held constant at 10:1 for all assays. (A) Kinetic traces of SRB fluorescence as a function of increasing pNP concentration. Release of 100% is the total amount of fluorescence obtained upon rupturing vesicles with detergent Triton-X-100. (B) Bar chart showing the percentage increase in SRB emission from (A)C) Kinetic traces of SRB fluorescence as a function of increasing pNP2 concentration. Release of 100% is defined as above. (D) Bar chart showing the percentage increase in SRB emission from (C)The data represent averages and standard deviations from at least 3 independent experiments.

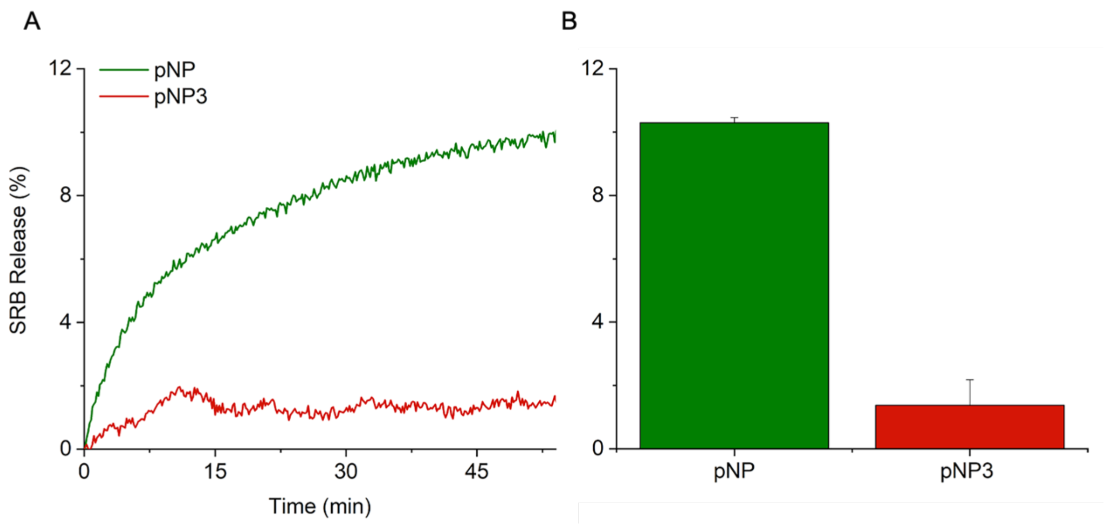

Figure S13. Comparison of SRB dye release from vesicles carrying pNP or pNP3.
The lid in pNP3 does not possess the TBA sequence making it unable to open in the presence of thrombin. pNP and pNP3 (30 µL, 1 µM) were added to vesicles containing 50 mM encapsulated SRB, which were then mixed with 2 µM thrombin. (A) Kinetic fluorescence traces of the dye release assay showing pNP (in green) pNP3 (red). (B) Bar chart of the net fluorescence increase after 50 min as illustrated in (A). The data represent averages and standard deviations from 3 independent experiments.

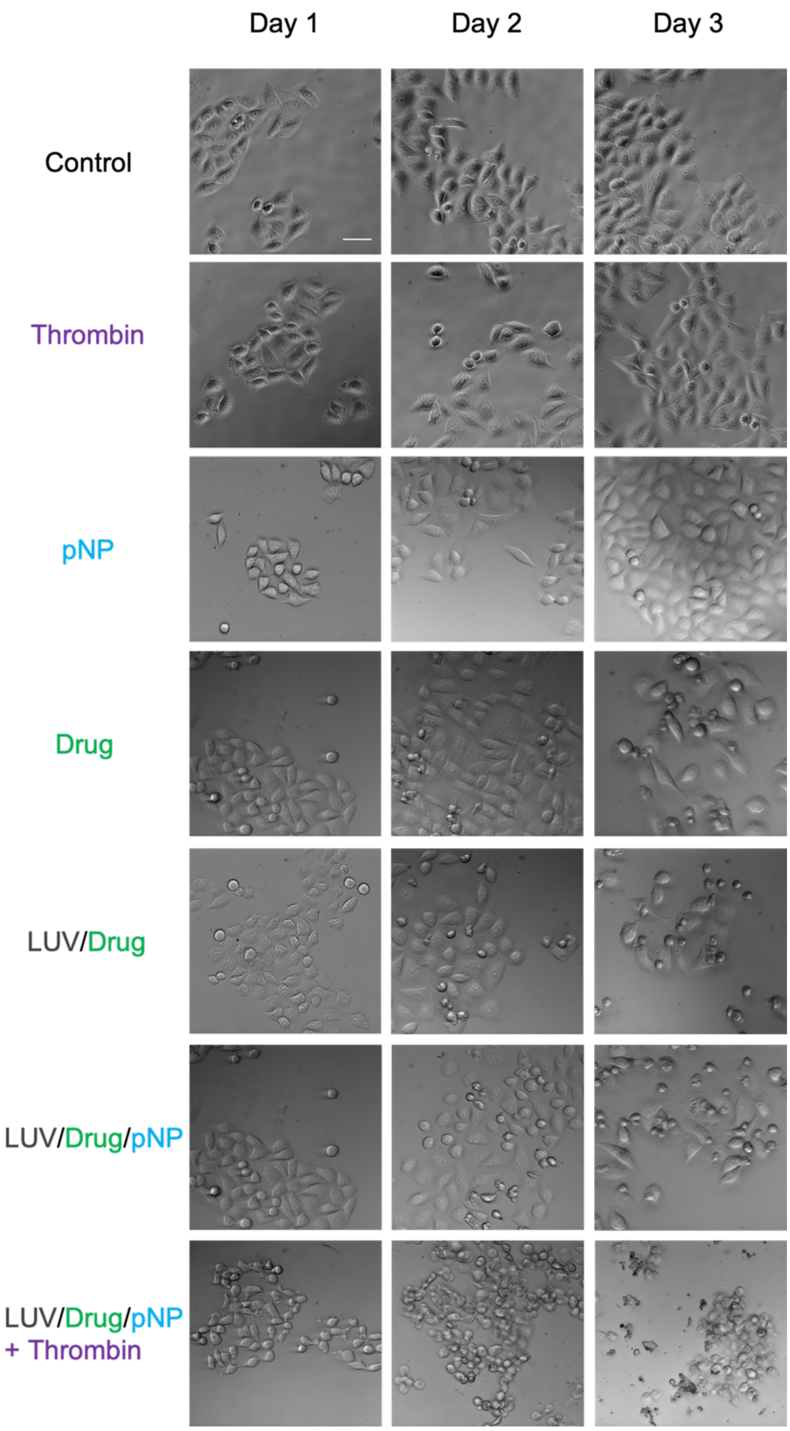

Figure S14. Brightfield images of HeLa cells, treated with different components of the nanodevice.

Representative images from 3 independent experiments are shown. Images were captured using a 20X air objective at 1, 2 and 3 days. To be consistent with the naming of the protein-gated pore in Fig. 4 of the paper, pNP in this illustration refers to pNP2. Scale bar, 50 µm.
